# Community-based care for autistic youth: community providers’ reported use of treatment practices in the United States

**DOI:** 10.3389/fpsyt.2023.1212084

**Published:** 2023-09-18

**Authors:** Amy Drahota, Aksheya Sridhar, Lauren J. Moskowitz, Connor M. Kerns, Latha Soorya, Allison Wainer, Elizabeth Cohn, Matthew D. Lerner

**Affiliations:** ^1^Department of Psychology, Michigan State University, East Lansing, MI, United States; ^2^Department of Psychology, St. John’s University, Queens, NY, United States; ^3^Department of Psychology, University British Columbia, Vancouver, BC, Canada; ^4^Department of Psychiatry and Behavioral Sciences, Rush University Medical Center, Chicago, IL, United States; ^5^School of Nursing, Hunter College, CUNY, New York, NY, United States; ^6^Department of Psychology, Stony Brook University, Stony Brook, NY, United States

**Keywords:** autism spectrum disorder, health services, community-based care services, transdisciplinary practice sets, community providers, generalized estimating equation (GEE), psychosocial treatment practices

## Abstract

**Introduction:**

To illustrate the landscape of community-based care for autistic youth in the United States, we identified transdisciplinary psychosocial intervention practice sets that community providers report utilizing to care for this population, and examined characteristics associated with provider-reported utilization.

**Methods:**

The Usual Care for Autism Study (UCAS) Survey assessed provider demographics and provider-reported use of transdisciplinary practices for common ASD co-occurring problems: social difficulties, externalizing behaviors, and anxiety. Community practitioners (*N* = 701) from allied health, behavioral, education, medical, mental health and other disciplines who treat or work with autistic youth (7–22 years) participated.

**Results:**

Exploratory factor analysis yielded four factors: Consequence-Based Strategies (CBS), Cognitive-Behavioral and Therapy Strategies (CBTS), Antecedent-Based Strategies (ABS), and Teaching Strategies (TS). Providers across disciplines reported utilizing ABS more often than other sets. Providers from behavioral disciplines, with less than 4-year or Master degrees, or with more experience reported the most use of ABS, CBS and CBTS. Medical and behavioral providers reported the most use of TS. Setting and child characteristics were associated with practice set use, indicating variability by disability and client socioeconomic status.

**Discussion:**

Findings reflect the complexity and inconsistency of the service landscape for autistic youth across the U.S. Only by understanding the service landscape and predictors of practice utilization, can researchers, policymakers, provider groups, and the autistic community facilitate effective implementation strategy development and use to ultimately improve community-based care.

## Introduction

Current prevalence estimates indicate that autism spectrum disorder (ASD) affects 1 in 36 children in the United States (1). Although social communication difficulties, including difficulties understanding how to initiate or maintain social interaction or social relationships with others, and restrictive and/or repetitive behaviors are the core impairments of ASD, co-occurring mental health problems are highly prevalent within this population, with anxiety and externalizing symptoms the most common and impairing (2, 3). Indeed, anxiety is one of the most common presenting problems for autistic individuals (4), with as many as 69% of autistic youth experiencing clinically significant anxiety disorders (5). Further, externalizing behaviors (including aggression, tantrums, self-injurious behaviors, noncompliance, and elopement) are also one of the most prevalent problems for this population, with 53% to 68% of autistic youth demonstrating aggression or tantrums (6–8). Resultingly, autistic youth have elevated service needs due to the chronicity and severity of ASD core symptoms and co-occurring mental health problems, particularly for youth co-presenting with social difficulties, externalizing behaviors, or anxiety symptoms (9, 10). However, there exists a well-known gap between intervention research and community-based practice for autistic youth and their families (11–16), particularly in community settings (e.g., community mental and behavioral health clinics, schools, private outpatient therapy or treatment clinics) providing services to school-age autistic youth (13, 17). Due to this gap, autistic youth experience high rates of unmet mental and behavioral health needs (18).

Several factors contribute to this research-to-practice gap, including a paucity of trained community-based providers; child, family and provider characteristics, and a lack of compatibility between interventions studied within research settings—that tend to focus on intervention efficacy—and community-based practice settings (11, 12, 15, 19). Indeed, community-based service organizations vary in terms of policy, organizational structure, and funding, which further contributes to challenges in translating interventions to these settings (12, 20) or understanding what the landscape of services entails within the U.S. e.g., which practices are being systematically used, underused, or mis-implemented; (21) and by whom (e.g., provider discipline, education, training, license, etc.). For example, autistic youth receive intervention services in many different settings (e.g., school, outpatient clinics in hospitals/medical centers, community mental health centers, research centers, private practice) and from providers representing a wide array of disciplines including behavior therapists, psychologists, social workers, educators, pediatricians, nurses, neurologists, and – most commonly – allied health professionals including speech language therapists and occupational therapists (22, 23). These disciplines have varied education levels, training backgrounds, and therapeutic orientations, may use different standards for evaluating the efficacy and effectiveness of practices, and may use different intervention practices and/or different terminology to describe those strategies, all of which result in differing approaches to routine or “usual” care found in the community (22). Further, this service landscape often results in siloed or fragmented care (22, 24, 25). In particular, the different terminology used by different disciplines can make communication across disciplines challenging, as they “speak different languages” (26, p. 374). This variability of professionals and service settings may lead to inconsistent implementation of practices for autistic youth and their families, and variable communication between providers within different systems of care; to date, even identifying such inconsistency has proven difficult due to the vast array of practices, practitioners, and settings (27, 28). Therefore, to begin to bridge the research-practice gap, a vital question remains underexamined: which individual intervention practices (or coherent groups thereof) are being used to treat autistic youth, by whom, and in what settings (i.e., what is the *landscape of practices in the United States*)? Understanding the practice landscape within community-based, usual care settings will allow us to promote a common vocabulary of treatment practices, facilitate improved communication and coordination of care, and enact effective implementation strategies to support evidence-based practice uptake and sustained use as well as de-implementation of nonevidence-based or unacceptable practices within this provider population (13, 20, 29, 30).

Implementation science (IS) provides methods for active, intentional efforts to embed clinical practices within organizations or service systems (31). A primary goal of IS is to maximize the fit, feasibility, acceptability, and utility of practices within implementation settings (32). Therefore, a comprehensive understanding of the existing service landscape (i.e., provider *use of* practices) for treating common mental and behavioral health concerns experienced by autistic youth is a critical first step that will lead to identifying and utilizing implementation strategies to improve the quality of ASD services in community-based settings (33). Moreover, it is crucial to first *identify* transdisciplinary treatment practices, defined as psychosocial treatment practices used by providers from two or more disciplines, in community-based settings regardless of empirical support status. Identification of transdisciplinary treatment practices are thought to: (1) increase the generalizability of effective implementation strategies (e.g., training protocols, fidelity measures, consistency across service settings) that facilitate the adoption and utilization of these practices, and (2) allow for more individualized, modular approaches to selection and use of these practices despite the discipline that developed the practice (34, 35). Empirically categorizing these discrete practices into groups of transdisciplinary “practice sets”—groups of practices that “hang together” such that when providers report utilizing one, they are likely to report utilizing others within the same set—can advance the field by elucidating patterns of community-based psychosocial care being provided to autistic youth by an array of practitioners. Thus, we sought to identify transdisciplinary sets of practices that community-based providers report using to treat autistic youth, report mean use rates of provider-reported practices sets, and analyze provider-, setting- and client-level characteristics predicting utilization of practice sets with autistic youth.

## Materials and methods

### Participants

We recruited U.S. community-based behavioral, educational, medical, mental health, allied health, and other providers treating social difficulties, externalizing behaviors, or anxiety in autistic youth 7–22 years old during the year prior to recruitment (36, 37). Participants were eligible if they completed a screening questionnaire indicating that they met the criteria above, provided an email address, and were located within 100 miles of a consortium member site (Chicago, Illinois; New York, New York; Philadelphia, Pennsylvania; San Diego, California; Long Island, New York). Recruitment methods included emailing professional listservs, professional networks, university alumni, and professional associations (e.g., Behavior Analyst Certification Board). Providers were encouraged to forward recruitment materials. Snowballing recruitment efforts and lack of the total number of ASD providers within eligible geographic areas prevented the estimation of response rate calculations. In total, 1,827 screening surveys were completed. Of these, 1,231 provider email addresses were supplied to an independent survey firm contracted to collect the online survey data. Of the providers recruited to participate, 701 (56.9%) completed the online Usual Care for Autism Study (UCAS) Survey. See Table 1 for provider demographics.

**Table 1:** Participant demographics.

	Total	Philadelphia, Pennsylvania	Chicago, Illinois	New York, New York	San Diego, California	Long Island, New York
	*N* (%)	*n* (%)	*n* (%)	*n* (%)	*n* (%)	*n* (%)
	672 (100%)	158 (23.4%)	166 (24.6%)	159 (23.6%)	129 (19.1%)	62 (9.2%)
Provider discipline						
Allied health^a^	146 (21.7%)	35 (22.2%)	35 (21.1%)	22 (13.8%)	38 (29.5%)	16 (25.8%)
Behavioral^b^	112 (16.6%)	24 (15.2%)	41 (24.7%)	16 (10.1%)	27 (20.9%)	4 (6.5%)
Education^c^	156 (23.1%)	30 (19%)	26 (15.7%)	73 (45.9%)	19 (14.7%)	8 (12.9%)
Medical^d^	63 (9.3%)	14 (8.9%)	30 (18.1%)	9 (5.7%)	7 (5.4%)	3 (4.8%)
Mental health^e^	126 (18.7%)	39 (24.7%)	22 (13.3%)	22 (13.8%)	19 (14.7%)	24 (38.7%)
Other^f^	71 (10.5%)	16 (10.1%)	12 (7.2%)	17 (10.7%)	19 (14.7%)	7 (11.3%)
Educational attainment						
<4-year degree	13 (1.9%)	3 (1.9%)	3 (1.8%)	2 (1.3)	3 (2.3%)	2 (3.2%)
4-year degree	78 (11.6%)	23 (14.6%)	29 (17.5%)	8 (5%)	13 (10.1%)	5 (8.1%)
Master’s degree	453 (67.3%)	95 (60.1%)	106 (63.9%)	117 (73.6%)	100 (77.5%)	35 (56.5%)
Doctoral degree	130 (19.3%)	37 (23.4%)	28 (16.9%)	32 (20.1%)	13 (10.1%)	20 (32.3%)
Years working with ASD						
0–10 years	364 (54%)	97 (61.4%)	78 (47%)	97 (61%)	65 (50.4%)	27 (43.5%)
11–20 years	247 (36.6%)	44 (27.8%)	80 (48.2%)	47 (29.6%)	53 (41.1%)	23 (37.1%)
20+ years	62 (9.2%)	17 (10.8%)	8 (4.8%)	15 (9.4%)	10 (7.8%)	12 (19.4%)
Missing	1 (0.1%)	0	0	0	1 (0.8%)	0
Treatment settings						
1 setting	434 (64.4%)	102 (64.6%)	95 (57.2%)	113 (71.1%)	85 (65.9%)	39 (62.9%)
2+ settings	240 (35.6%)	56 (35.4%)	71 (42.8%)	46 (28.9%)	44 (34.1%)	23 (37.1%)
Treat co-occurring IDD						
Unsure/Do not know	25 (3.7%)	4 (2.5%)	3 (1.8%)	13 (8.2%)	5 (3.9%)	0
Never/Rarely	54 (8.0%)	13 (8.2%)	9 (5.4%)	16 (10.1%)	8 (6.2%)	8 (12.9%)
Sometimes	348 (51.6%)	88 (55.7%)	100 (60.2%)	65 (40.9%)	69 (53.5%)	26 (41.9%)
Frequently	247 (36.6%)	53 (33.5%)	54 (32.5%)	65 (40.9%)	47 (36.4%)	28 (45.2%)
High SES						
No	414 (61.4%)	101 (63.9%)	110 (66.3%)	103 (64.8%)	60 (46.5%)	40 (64.5%)
Yes	260 (38.6%)	57 (36.1%)	56 (33.7%)	56 (35.2%)	69 (53.5%)	22 (35.5%)
Low SES						
No	297 (44.1%)	48 (30.4%)	90 (54.2%)	77 (48.4%)	59 (45.7%)	23 (37.1%)
Yes	377 (55.9%)	110 (69.6%)	76 (45.8%)	82 (51.6%)	70 (54.3%)	39 (62.9%)

a Comprised of speech and language therapists, occupational therapists, and physical therapists.

b Comprised of behavioral therapists, behavioral analysts, behavioral technicians, etc.

c Comprised of special educators, general educators, school psychologists, etc.

d Comprised of psychiatrists, neurologists, health practitioners, etc.

e Comprised of social workers, psychologists, counselors, etc.

f Comprised of managers, administrators, support workers, other disciplines, multiple disciplines, and unknown.

### Procedure

Each consortium site obtained institutional IRB approval for study procedures and recruited participants. Eligible participants were emailed a unique URL to access the web-based survey by Princeton Survey Research Associates International (PSRAI). The first page of the online survey included the IRB-approved consent form. Participants consented to participate then advanced to the survey. Respondents completing the survey were paid a $40 honorarium. Data were de-identified by PSRAI and provided to the UCAS consortium members for analysis.

### Measure

**Usual Care for Autism Study (UCAS) Survey** (38). Development of the UCAS Survey included a 2-round Delphi poll, involving expert community-based ASD providers representing disciplines who often serve autistic youth (*cf.* 22), to ensure transdisciplinary construct validity (e.g., reduced discipline-specific jargon, definitions of practices, etc.) (38, 39).

The survey included demographic questions and an inventory of discrete ASD treatment practices. Demographic items included provider- (discipline, educational attainment, years providing ASD services), treatment- (number of treatment settings), and client-level [co-occurring intellectual/developmental disability (IDD), socioeconomic status (SES)] characteristics. Of the 55 practices that comprised the full UCAS survey inventory, we focused on a subset of 32 practices that were endorsed, based on expert consensus (38), as treatments for all three of the identified key treatment areas: social challenges, externalizing symptoms, and anxiety. As in Lerner et al. (40), we did not include the remaining 23 practices in the analysis for this paper since they were identified by experts as specific (e.g., endorsed by experts in treating anxiety only), rather than transdiagnostic (endorsed for treating all 3 presenting problems). We included only these 32 transdiagnostic strategies because the purpose of the present study was to focus on coherent sets of intervention practices that are used by providers to treat autistic youth regardless of the treatment area. As Lerner et al. (40) noted in a previous study involving transdiagnostic practices on the UCAS, “If a practice was identified to be specific only to the treatment of anxiety, for instance, this would likely skew its relation to other identified practices.” Thus, including only these 32 transdiagnostic practices allowed us to identify the practices sets likely to be most generalized to all types of providers who serve autistic youth (40). First, participants rated their familiarity with each practice on a 4-point Likert scale (1 = *never heard of/not at all familiar*; 4 = *very familiar*) (40). Providers who were *somewhat* or *very familiar* with a practice were then asked to report how often they utilized the practice to treat autistic youth in the past year (1 = *not at all*, 4 = *very commonly*).

### Analysis

#### Data screening

The data structure was examined to ensure variables met assumptions for the planned analyses. Further, 27 participants were flagged by PSRAI due to concerns about suspicious or fraudulent participation (41). *T*-test comparisons between flagged and unflagged participant responses were statistically significant (*p*’s < 0.05). Thus, 674 provider responses were included in the data analyses. Of note, given the variability in providers’ familiarity with these practices, sample sizes varied for each practice set, ranging from 119–350 (42).

#### Factor analysis

Exploratory factor analysis using weighted least squares means and variance adjusted estimation (WLSMV) and goemin rotation were run in M+ to identify converging practice sets. Factors with eigenvalues greater than one were retained. These practice sets were used to examine raw differences in reported mean use by demographic variable, and as dependent variables in the comparative analyses.

#### Generalized estimating equations

Comparative analyses were conducted utilizing GEEs. GEEs—able to account for possible non-independence of participant responses—identified provider-, setting- and client-level characteristics significantly associated with provider-reported use of practice sets (43, 44). Controlling for site differences, we predicted reported use of practice sets (DVs) by the following independent variables: provider discipline, educational attainment, number of years working with autistic youth, number of treatment settings, and child characteristics, including co-occurring IDD, and high and low SES. We ran each GEE analysis with unstructured and independent model structures, selecting the model with the lowest QIC model criterion coefficient.

## Results

Providers from behavioral disciplines self-reported utilizing the most strategies overall, followed by those from the “other” discipline, then allied health, medical, educational, and mental health professions. Lower educational attainment (<4-year degree) and low- to mid-range experience (0–10 years, 11–20 years) were associated with the most reported strategy use across all strategies. Providers working in two or more treatment settings (referred to as *settings*) reported greater overall use of strategies than those working in a single setting. Providers who reported *sometimes* working with autistic youth with co-occurring IDD endorsed the greatest variety of strategies utilized. Working with children from high SES backgrounds was associated with lower overall use of strategies, while no difference in average use of strategies was associated with working with youth from low SES backgrounds.

### Factor analysis

EFA revealed four transdisciplinary practice sets, with loading patterns that accounted for 75% of the variance in item responses (Table 2). Factor one, *Consequence-Based Strategies* (CBS; 8 items; *n* = 287), had a mean use rating of 3.42 (SD = 0.61). Factor two, *Cognitive-Behavioral and Therapy Strategies* (CBTS; 12 items; *n* = 119), had a mean use rating of 3.16 (SD = 0.57). Factor three, *Antecedent-Based Strategies* (ABS; 8 items; *n* = 350), had a mean use rating of 3.48 (SD = 0.46). Factor four, *Teaching Strategies* (TS; 4 items; *n* = 266), had a mean use rating of 3.15 (SD = 0.71). Provider-reported mean practice set use indicated that providers utilize ABS most often, followed by CBS, CBTS, and TS, respectively. Table 3 provides mean reported strategy use and practice sets.

**Table 2:** Factor loadings from exploratory confirmatory factor analysis of use practice sets.

	Factor loadings	Factor loadings	factor loadings	Factor loadings
Use practice set (1) consequence-based strategies (CBS; 8 items)				
Differential reinforcement	0.688*	0.019	0.061	0.059
Extinction	0.678*	0.141	0.082	−0.115
Functional behavioral assessment (FBA)	0.552*	−0.051	0.024	0.239
Functional communication training (FCT)	0.465*	−0.06	0.314*	0.161
Noncontingent reinforcement or built-in breaks	0.390*	0.009	0.023	0.334
Positive reinforcement	0.827*	0.066	0.003	0.11
Reinforcement schedules	0.640*	0.001	−0.033	0.086
Token economy	0.712*	0.02	−0.04	0.254
Use practice set (2) cognitive-behavioral and therapy strategies (CBTS; 12 items)				
Didactic training, social scripts, instructional learning	0.081	0.326*	0.262*	0.086
Games, activities that require social interaction skills	−0.096	0.377*	0.16	0.254
Gradual, graded or habituated exposure/systematic desensitization	0.109	0.526*	−0.222*	0.285*
Homework	−0.099	0.574*	0.053	−0.069
Motivation by incorporating special interests into activities	0.047	0.393*	0.284*	0.059
Parent coaching	0.073	0.424*	0.135	0.182
Performance feedback	0.139	0.440*	0.046	0.073
Priming	0.075	0.423*	0.188*	0.008
Psychoeducation	0.014	0.889*	−0.423*	−0.015
Self-awareness of bodily responses	−0.005	0.801*	−0.028	−0.098
Self-management	0.008	0.456*	−0.048	0.348*
Socratic discussions	−0.079	0.548*	0.055	0.119
Use practice set (3) antecedent-based strategies (ABS; 8 items)				
Choice Making/ Providing Choices	0.269*	0.206*	0.380*	−0.142
Embedding special interests in social interaction	−0.048	0.245*	0.545*	−0.025
Environmental structuring	0.388*	0.025	0.473*	−0.142
Modeling or imitation	0.232*	0.216*	0.354*	0.006
Prompt fading	0.198	−0.017	0.420*	0.382
Prompting	0.255	−0.074	0.475*	0.287
Stories/vignettes	0.061	0.270*	0.286*	0.19
Visual tools or supports	0.154	0.194	0.482*	0.112
Use practice set (4) teaching strategies (TS; 4 items)				
Peer modeling or peer mentoring	−0.027	0.257*	0.171	0.438*
Shaping	0.184	0.143	0.123	0.511*
Stimulus control	0.349	0.104	−0.02	0.454*
Task analysis/chaining	−0.068	0.19	0.348	0.437*
**% variance**	**51%**	**11%**	**7%**	**6%**

% variance represents the proportion of the variance across all modeled items explained by the given component.

**Table 3:** Mean use scores by demographic variables.

	Reported use total	Practice set 1: CBS	Practice set 2: CBTS	Practice set 3: ABS	Practice set 4: TS
	*N* = 674	*n* = 287	*n* = 119	*n* = 350	*n* = 266
	Mean (SD)	Mean (SD)	Mean (SD)	Mean (SD)	Mean (SD)
Mean use scores	3.19 (0.53)	3.42 (0.61)	3.16 (0.57)	3.48 (0.46)	3.15 (0.71)
Provider discipline					
Allied health	3.17 (0.46)	3.13 (0.59)	2.83 (0.53)	3.48 (0.40)	3.00 (0.60)
Behavioral	3.47 (0.38)	3.76 (0.34)	3.59 (0.22)	3.66 (0.36)	3.47 (0.54)
Education	3.11 (0.53)	3.43 (0.59)	2.99 (0.51)	3.36 (0.52)	2.99 (0.72)
Medical	3.13 (0.73)	3.11 (0.62)	3.33 (0.38)	3.27 (0.34)	3.21 (0.27)
Mental health	3.06 (0.54)	3.16 (0.76)	3.36 (0.62)	3.39 (0.56)	2.77 (0.93)
Other	3.28 (0.51)	3.54 (0.50)	3.09 (0.54)	3.57 (0.40)	3.28 (0.67)
Educational attainment					
<4-year degree	3.39 (0.64)	3.78 (0.27)	3.33 (*n* = 1)	3.63 (0.36)	3.31 (0.85)
4-year degree	3.28 (0.49)	3.46 (0.72)	3.04 (0.46)	3.55 (0.45)	3.37 (0.56)
Master’s degree	3.20 (0.51)	3.46 (0.57)	3.16 (0.60)	3.51 (0.44)	3.23 (0.66)
Doctoral degree	3.10 (0.62)	3.21 (0.68)	3.16 (0.55)	3.25 (0.54)	2.63 (0.77)
Years working with ASD					
0–10 years	3.20 (0.52)	3.51 (0.53)	3.15 (0.64)	3.55 (0.40)	3.19 (0.65)
11–20 years	3.21 (0.55)	3.33 (0.65)	3.08 (0.44)	3.34 (0.54)	3.08 (0.77)
21+	3.11 (0.56)	3.27 (0.76)	3.31 (0.49)	3.49 (0.50)	3.10 (0.76)
Treatment settings					
1 setting	3.13 (0.52)	3.42 (0.55)	3.19 (0.58)	3.47 (0.47)	3.07 (0.74)
2+ settings	3.31 (0.54)	3.42 (0.69)	3.11 (0.57)	3.49 (0.46)	3.25 (0.64)
Treat co-occurring IDD					
Unsure/do not know	2.99 (0.73)	3.43 (0.85)	2.58 (*n* = 1)	3.44 (0.73)	2.83 (1.10)
Never/rarely	3.04 (0.47)	3.27 (0.73)	3.03 (0.73)	3.43 (0.41)	2.81 (0.82)
Sometimes	3.23 (0.53)	3.40 (0.63)	3.17 (0.62)	3.47 (0.48)	3.14 (0.71)
Frequently	3.18 (0.51)	3.47 (0.55)	3.17 (0.49)	3.50 (0.43)	3.22 (0.64)
High SES					
No	3.20 (0.53)	3.45 (0.57)	3.04 (0.60)	3.47 (0.47)	3.09 (0.73)
Yes	3.18 (0.53)	3.38 (0.66)	3.28 (0.52)	3.49 (0.47)	3.21 (0.67)
Low SES					
No	3.19 (0.55)	3.36 (0.69)	3.07 (0.60)	3.40 (0.50)	3.08 (0.77)
Yes	3.19 (0.52)	3.45 (0.56)	3.21 (0.55)	3.52 (0.44)	3.18 (0.67)

Use scores range from 1 = not at all to 4 = very commonly.

### Comparative GEE analyses

Omnibus GEEs indicated that provider-, setting-, and client-level characteristics predicted provider-reported use of each practice set. *Post hoc* analyses are presented below and in Table 4.

**Table 4:** Provider-reported use of practice sets.

**Practice set (1) consequence-based strategies (CBS)**				
Unstructured GEE correlation model structure utilized (QIC = 117.57)				
**Provider *n* = 287**	**χ**^2^**/Mean**	**S.E.**	**95% C.I.**	**Contrasts (*p*’s < 0.05)**
***Provider-level characteristics***				
**Provider discipline**	2477.22***			
Allied health^a^	2.70	0.095	2.51–3.88	*a*, < *b*, *c*, *f*
Behavioral^b^	3.40	0.063	3.27–3.52	*a*, *c*, *d*, *e*, *f* < *b*
Education^c^	2.95	0.072	2.80–3.09	*a*, *e* < *c* < *b*, *f*
Medical^d^	2.84	0.116	2.61–3.07	*d*, < *b*, *f*
Mental health^e^	2.70	0.087	2.53–2.87	*e*, < *b*, *c*, *f*
Other^f^	3.25	0.057	3.14–3.36	*a*, *c*, *d*, *e* < *f* < *b*
**Educational attainment**	70.14***			
<4-year degree^g^	3.17	0.082	3.01–3.33	*h*, *j* < *g*
4-year degree^h^	2.71	0.126	2.47–2.96	*h* < *g*, *i*
Master’s degree^i^	3.09	0.047	3.00–3.19	*h*, *j* < *i*
Doctoral degree^j^	2.90	0.037	2.83–2.97	*j* < *g*, *i*
**Years working with ASD**	49.90***			
0–10 years^k^	2.83	0.046	2.74–2.92	*k* < *l*, *m*
11–20 years^l^	3.03	0.070	2.89–3.17	*k* < *l*
21+^m^	3.05	0.071	2.91–3.19	*k* < *m*
***Setting-level characteristic***				
**Treatment settings**	7.61**			
1 setting	3.05	0.065	2.93–3.18	2+ settings <1
2+ settings	2.89	0.070	2.75–3.02	
***Child-level characteristics***				
**Treat co-occurring IDD**	129.19**			
Unsure/do not know^n^	2.62	0.095	2.44–2.81	*n* < *o*, *p*, *q*
Never/rarely^o^	2.85	0.083	2.69–3.01	*n* < *o* < *p*, *q*
Sometimes^p^	3.20	0.079	3.04–3.35	*n*, *o* < *p*
Frequently^q^	3.21	0.040	3.14–3.29	*n*, *o* < *q*
**High SES**	16.71***			
No	3.05	0.055	2.94–3.16	Yes < No
Yes	2.89	0.071	2.75–3.03	
**Low SES**	8.99**			
No	2.90	0.078	2.75–3.05	No < Yes
Yes	3.04	0.048	2.95–3.13	
**Practice set (2) cognitive-behavioral and therapy strategies (CBTS)**				
Unstructured GEE correlation model structure utilized (QIC = 37.32)				
**Provider *n* = 119**	**χ**^2^**/ Mean**	**S.E.**	**95% C.I.**	**Contrasts** (*p*’s < 0.05)
***Provider-level characteristics***				
**Provider discipline**	**2.06**^12^***			
Allied health^a^	2.96	0.071	2.82–3.10	***a*** < *b*, *d*, *e*, *f*
Behavioral^b^	3.43	0.077	3.27–3.58	*a*, *c*, *e*, *f* < *b*
Education^c^	3.03	0.094	2.85–3.22	*c* < *b*, *d*, *e*, *f*
Medical^d^	3.34	0.069	3.20–3.47	*a*, *c*, *f* < *d*
Mental health^e^	3.23	0.063	3.11–3.35	*a*, *c*, *f* <*e* < *b*
Other^f^	3.15	0.078	3.00–3.30	*a*, *c* < *f* < *b*, *d*, *e*
**Educational attainment**	49.22***			
<4-year degree^g^	3.25	0.055	3.15–3.36	*j* < *g*
4-year degree^h^	3.17	0.129	2.92–3.43	*NS*
Master’s degree^i^	3.26	0.044	3.17–3.34	*j* < *i*
Doctoral degree^j^	3.07	0.045	2.99–3.16	*j* < *g*, *i*
**Years working with ASD**	23.40***			
0–10 years^k^	3.08	0.049	2.98–3.18	*k* < *l*, *m*
11–20 years^l^	3.17	0.074	3.03–3.32	*k* < *l*
21+^m^	3.32	0.090	3.14–3.49	*k* < *m*
***Setting-level characteristic***				
**Treatment settings**	11.65***			
1 setting	3.23	0.060	3.12–3.35	2+ settings <1
2+ settings	3.15	0.068	3.01–3.28	
***Child-level characteristics***				
**Treat co-occurring IDD**	22.63***			
Unsure/do not know^n^	3.36	0.131	3.11–3.62	*q* < *n*
Never/rarely^o^	3.16	0.134	2.89–3.42	*NS*
Sometimes^p^	3.17	0.028	3.12–3.22	*q* < *p*
Frequently^q^	3.07	0.038	3.00–3.14	*q* < *n*, *p*
**High SES**	68.18***			
No	3.10	0.068	2.97–3.23	No < Yes
Yes	3.28	0.06	3.16–3.40	
**Low SES**	7.71**			
No	3.12	0.053	3.02–3.23	No < Yes
Yes	3.26	0.079	3.10–3.41	
**Practice set (3) antecedent-based strategies (ABS)**				
Unstructured correlation model structure utilized (QIC = 82.85)				
**Provider *n* = 350**	**χ**^2^**/ Mean**	**S.E.**	**95% C.I.**	**Contrasts** (*p*’s < 0.05)
***Provider-level characteristics***				
**Provider discipline**	**3.18**^12^***			
Allied health^a^	3.35	0.041	3.26–3.43	*c*, *d*, *e* < *a* < *b*
Behavioral^b^	3.50	0.047	3.41–3.60	*a*, *c*, *d*, *e*, *f* < *b*
Education^c^	3.28	0.028	3.22–3.33	*c* < *a*, *b*, *f*
Medical^d^	3.28	0.056	3.17–3.39	*d* < *b*, *f*
Mental health^e^	3.26	0.048	3.17–3.36	*e* < *a*, *b*, *f*
Other^f^	3.41	0.028	3.35–3.46	*c*, *d*, *e* < *f* < *b*
**Educational attainment**	78.54***			
<4-year degree^g^	3.61	0.071	3.47–3.75	*h*, *i*, *j* < *g*
4-year degree^h^	3.26	0.071	3.12–3.40	*h* < *g*
Master’s degree^i^	3.33	0.021	3.29–3.37	*j* < *i* < *g*
Doctoral degree^j^	3.19	0.028	3.13–3.24	*j* < *g*, *i*
**Years working with ASD**	86.41***			
0–10 years^k^	3.32	0.030	3.26–3.38	*l* < *k*< *m*
11–20 years^l^	3.25	0.033	3.18–3.31	*l* < *k* < *m*
21+^m^	3.47	0.046	3.37–3.56	*l* < *k* < *m*
***Setting-level characteristic***				
**Treatment settings**	16.35***			
1 setting	3.37	0.031	3.31–3.43	2+ settings <1
2+ settings	3.32	0.037	3.25–3.39	
***Child-level characteristics***				
**Treat co-occurring IDD**	61.71***			
Unsure/Do not know^n^	3.18	0.059	3.07–3.30	*n* < *o*, *p*, *q*
Never/Rarely^o^	3.39	0.016	3.35–3.41	*n* < *o* < *q*
Sometimes^p^	3.36	0.041	3.28–3.44	*n* < *p* < *q*
Frequently^q^	3.45	0.035	3.38–3.52	*n*, *o*, *p* < *q*
**High SES**	0.73			
No	3.34	0.036	3.27–3.41	
Yes	3.35	0.032	3.28–3.41	
**Low SES**	4.30*			
No	3.33	0.037	3.26–3.40	No < Yes
Yes	3.36	0.031	3.30–3.42	
**Practice set (4) teaching strategies (TS)**				
Unstructured correlation model structure utilized (QIC = 120.36)				
**Provider *n* = 266**	**χ**^2^**/ Mean**	**S.E.**	**95% C.I.**	**Contrasts** (*p*’s < 0.05)
***Provider-level characteristics***				
**Provider discipline**	**1.558**^11^***			
Allied health^a^	2.83	0.072	2.69–2.97	*a* < *b*, *c*, *d*, *e*, *f*
Behavioral^b^	3.25	0.051	3.14–3.35	*a*, *c*, *e* < *b*
Education^c^	3.11	0.035	3.04–3.18	*a* < *c* < *b*, *d*
Medical^d^	3.34	0.062	3.22–3.46	*a*, *c*, *e*, *f* < *d*
Mental health^e^	2.96	0.106	2.75–3.17	*a* < *e* < *b*, *d*, *f*
Other^f^	3.24	0.071	3.10–3.38	*a*, *e* < *f* < *d*
**Educational attainment**	1177.06***			
<4-year degree^g^	3.22	0.178	2.87–3.57	*j* < *g*
4-year degree^h^	3.38	0.072	3.23–3.52	*j* < *h*
Master’s degree^i^	3.39	0.034	3.32–3.45	*j* < *i*
Doctoral degree^j^	2.51	0.034	2.44–2.57	*j* < *g*, *h*, *i*
**Years working with ASD**	4.98			
0–10 years^k^	3.17	0.044	3.09–3.26	
11–20 years^l^	3.05	0.079	2.90–3.20	
21+^m^	3.15	0.047	3.05–3.24	
***Setting-level characteristic***				
**Treatment settings**	23.37***			
1 setting	3.00	0.043	2.91–3.08	1 setting <2+
2+ settings	3.25	0.070	3.11–3.38	
***Child-level characteristics***				
**Treat co-occurring IDD**	11.48**			
Unsure/do not know^n^	3.23	0.072	3.09–3.38	*o* < *n*
Never/rarely^o^	2.97	0.076	2.82–3.12	*o* < *n*, *q*
Sometimes^p^	3.12	0.068	2.99–3.26	*NS*
Frequently^q^	3.16	0.048	3.07–3.26	*o* < *q*
**High SES**	1.31			
No	3.11	0.045	3.02–3.20	
Yes	3.13	0.060	3.02–3.25	
**Low SES**	11.36**			
No	3.18	0.061	3.06–3.30	Yes < No
Yes	3.07	0.046	2.98–3.16	

*p < 0.05. **p < 0.01. ***p < 0.001. NS, non-significant. Use scores range from 1 = not at all to 4 = very commonly.

^a^Comprised of speech and language therapists, occupational therapists, and physical therapists.

^b^Comprised of behavioral therapists, behavioral analysts, behavioral technicians, etc.

^c^Comprised of special educators, general educators, school psychologists, etc.

^d^Comprised of psychiatrists, neurologists, health practitioners, etc.

^e^Comprised of social workers, psychologists, counselors, etc.

^f^Comprised of managers, administrators, support workers, other disciplines, multiple disciplines, and unknown.

#### Consequence-based strategies

Of the 287 providers who reported utilizing CBS, *post hoc* analyses found that providers from behavioral disciplines reported using CBS the most, and providers from allied and mental health disciplines reported using CBS the least (*p*’s < 0.001). Greatest reported use of CBS was associated with less than 4-year and Master degrees, compared with providers with bachelors or doctoral degrees (*p*’s < 0.01). Greater experience (11–20, 21+ years) was associated with most reported use of CBS strategies compared with less experience (*p*’s < 0.001). Providers working in a single setting reported greater use of CBS than those working in multiple settings (*p* = 0.006). Providers reported being more likely to deliver CBS if they frequently or sometimes work with autistic clients with co-occurring IDD than those who never/rarely or were unsure (*p*’s < 0.001). Providers also reported greater likelihood of delivering CBS if they did not treat clients from high SES or if they treated clients from low SES backgrounds compared with providers who do not treat clients from these specific SES backgrounds (*p*’s < 0.01).

#### Cognitive-behavioral and therapeutic strategies

Among providers who reported utilizing CBTS (*n* = 119), behavioral and medical providers reported the most use compared to other disciplines (*p*’s < 0.001), followed by mental health providers, who reported greater use of CBTS than allied health, education, and other providers (*p*’s < 0.05). Providers with less than 4-year or master’s degrees reported using CBTS more so than providers with doctoral degrees (*p*’s < 0.001). Additionally, providers with more experience (11–20 years, 21+ years) reported utilizing CBTS more than providers with less experience (*p*’s < 0.001). Providers working in a single setting reported more use of CBTS than providers working in multiple settings (*p* = 0.001). Providers who were unsure or did not know if their clients had co-occurring IDD reported the most use of CBTS while providers who frequently delivered services to autistic youth and co-occurring IDD were the least likely to report using CBTS (*p* < 0.05). Finally, providers treating autistic youth from high and low SES reported more use of CBTS than providers not treating autistic youth from these socioeconomic backgrounds (*p*’s < 0.01).

#### Antecedent-based strategies

Of the 350 providers who reported utilizing ABS, *post hoc* analyses found that providers from behavioral disciplines reported most use compared to all other groups (*p*’s < 0.001). Providers with less than 4-year degrees reported using ABS more than providers with other educational attainment (*p*’s < 0.001). Providers with the most years of experience reported the most use of ABS (*p*’s ≤ 0.001), followed by providers with 0–10 years of experience (*p*’s < 0.001). Providers working in a single setting reported more use of ABS than providers working in multiple settings (*p* < 0.001). Finally, providers who frequently delivered services to autistic youth and co-occurring IDD and providers who treating autistic youth from low SES backgrounds were more likely to deliver ABS (*p*’s ≤ 0.05) in contrast to comparison groups.

#### Teaching strategies

Among providers who reported utilizing TS (*n* = 266), *post hoc* analyses found medical providers reported more use of TS than other disciplines except for behavioral providers (*p*’s ≤ 0.01). Providers with doctoral degrees reportedly delivered TS less than other educational attainment categories (*p*’s < 0.001). Providers working in multiple settings reported more use of TS than providers working in a single setting (*p* < 0.001). Additionally, providers who did not know or frequently delivered services to autistic youth with co-occurring IDD were more likely to reportedly utilize TS than providers who never or rarely worked with autistic youth with IDD (*p*’s ≤ 0.05). Finally, providers who delivered services to autistic youth from low SES backgrounds were less likely to deliver TS as compared with providers treating youth who were not from low SES backgrounds (*p* = 0.001).

## Discussion

Our study provides the first large-scale, comprehensive evaluation of the landscape of self-reported practices utilized by community-based providers for autistic youth in community settings in the United States. Further, this study advances the field by identifying transdisciplinary practices commonly utilized together in community-based settings for autistic youth (see Table 2). Provider-, setting-, and client-level characteristics significantly predicted provider-reported use of strategies and specific practice sets. Importantly, these findings reflect the complexity, inconsistency, and confounding state of the landscape of treatment practices currently being delivered to autistic youth who experience social difficulties, externalizing behaviors, and/or anxiety (Table 5).

**Table 5:** Overall patterns of provider-reported practice set use.

**Provider discipline**	
Practice set 1: CBS	Behavioral > Other > Education = Medical > Mental health = Allied health
Practice set 2: CBTS	Behavioral = Medical, Medical = Mental health > Other > Education > Allied health
Practice set 3: ABS	Behavioral > Other = Allied health > Medical = Education = Mental health
Practice set 4: TS	Medical = Behavioral, Behavioral = Other, Other = Education > Mental health > Allied health
**Educational attainment**	
Practice set 1: CBS	Less than 4-year = Master’s > 4-year degree = Doctoral
Practice set 2: CBTS	Master’s = Less than 4-year* > Doctoral
Practice set 3: ABS	Less than 4-year degree > Master’s = 4-year degree > Doctoral
Practice set 4: TS	Master’s = 4-year degree = Less than 4-year > Doctoral
**Years working with ASD**	
Practice set 1: CBS	21+ years = 11–20 years >0–10 years
Practice set 2: CBTS	21+ years = 11–20 years >0–10 years
Practice set 3: ABS	21+ years >11–20 years >0–10 years
Practice set 4: TS	*NS*
**Treatment settings**	
Practice set 1: CBS	1 > 2+ setting
Practice set 2: CBTS	1 > 2+ setting
Practice set 3: ABS	1 > 2+ setting
Practice set 4: TS	2+ > 1 setting
**Co-occurring IDD**	
Practice set 1: CBS	Frequently = Sometimes > Never/Rarely > Unsure/Do not know
Practice set 2: CBTS	Unsure/Do not know = Sometimes > Frequently
Practice set 3: ABS	Frequently > Never/Rarely = Sometimes > Unsure/Do not know
Practice set 4: TS	Unsure/Do not know = Frequently > Never/Rarely
**High SES**	
Practice set 1: CBS	Not high SES > High SES
Practice set 2: CBTS	High SES > Not high SES
Practice set 3: ABS	*NS*
Practice set 4: TS	*NS*
**Low SES**	
Practice set 1: CBS	Low SES > Not low SES
Practice set 2: CBTS	Low SES > Not low SES
Practice set 3: ABS	Low SES > Not low SES
Practice set 4: TS	Not low SES > Low SES

*Only 1 respondent for this group. NS, No significant statistical difference.

Overall, providers who reported being somewhat or very familiar with the practices reported using ABS (e.g., providing choices) most and TS (e.g., peer modeling) least often. This may be because it is easier to use ABS, such as embedding special interests in interactions and environmental structuring, than to teach skills using procedures such as shaping or chaining. Notably, CBTS were reported to be utilized only slightly more than TS. However, the number of providers who were able to provide use ratings for CBTS was much lower than TS due to limited familiarity of the discrete practices comprising the CBTS practice set (*n* = 119 versus 266, respectively). Additionally, patterns of provider-reported practice set use was more variable for child-level characteristics than provider- or setting-level characteristics. Providers who frequently delivered treatment to autistic youth with co-occurring IDD used behavioral strategies–CBS and ABS–more than other providers. This finding is consistent with existing literature; cognitive components of cognitive-behavioral therapy are often reduced or eliminated when delivered to children with IDD (45, 46) despite adapted CBT being found to reduce anxiety symptoms in this population (47). Further, high and low SES was variably related to provider-reported practice set use.

### Provider and setting-level characteristics

Providers from behavioral disciplines, when familiar with these practices, reported using three of the four practice sets the most (ABS, CBTS, and CBS), relative to the other disciplines. Although medical and behavioral providers reported the most use of TS, this finding was not significantly different from other provider groups. While behavioral providers may have reported using ABS and CBS most commonly due to training or setting selection, an alternative explanation may be that behavioral providers are simply implementing more practices than other disciplines (22). Additionally, the finding that medical providers utilize TS at greater rates than other providers is surprising because existing literature remains equivocal. For example, Christon et al. (22) found that doctors and nurses provided significantly fewer evidence-based intervention practices, while Olfson et al. (48) found the rate of mental health services delivered to children during office visits has increased over time. Additional investigation into the practices delivered to autistic youth by medical providers and how they may relate to typically limited duration of medical visits would further elucidate these results.

Interestingly, providers with less than 4-year degrees reported the most use of all practice sets. This could be because paraprofessionals such as Registered Behavior Technicians (who need a high school diploma and 40 training hours) are typically the frontline direct-service providers implementing various instructional/skill acquisition and externalizing behavior reduction programs for autistic youth (49). Alternatively, providers with less education may have a lower threshold for endorsing the use of a strategy than those with greater training, regardless of whether they are *actually* utilizing them (50). Importantly, research highlights the need for intensive interventions delivered with high fidelity and quality for autistic youth; however, these findings suggest that providers with the least amount of education and training may be providing more varied interventions. Moreover, providers with higher educational levels may have developed practice specializations, thereby reducing the likelihood of endorsing the use of a broad range of practices. Nonetheless, working longer in the field of ASD was also associated with greater reported use of these practice sets, except for TS. This may be because teaching replacement behaviors/skills can be more difficult to implement (e.g., requiring more time, effort, and preparation) than other strategies (51), despite the effectiveness of active teaching strategies to support autistic youth. It may be that greater experience leads to an increased likelihood of utilizing many strategies, given increased familiarity of varied practices. Finally, for CBS, CBTS, and ABS, working in a single treatment setting was associated with greater reported use as compared with providers working in multiple treatment settings.

### Child characteristics

Child-level characteristics, including co-occurring IDD and socioeconomic background, predicted providers’ reported use of practice sets. Consistent with existing research on reducing externalizing behaviors (52), reducing anxiety (53) and improving social skills (54) in autistic individuals with co-occurring IDD, providers who sometimes or frequently worked with this population often reported using ABS and CBS. Providers who were unsure or did not know whether they treated autistic clients with co-occurring IDD were most likely to report utilizing CBTS. Limitations in the existing literature may explain this result; ASD research utilizing CBTS have historically involved autistic youth without IDD, which may influence intervention practices utilized for autistic youth with co-occurring IDD (55). Finally, providers who frequently or were unsure whether they delivered services to autistic youth with co-occurring IDD reported utilizing TS more often than providers who endorsed never or rarely delivering services to this population.

Lastly, providers familiar with CBTS were more likely to use them when they endorsed delivering services to autistic youth from both high and low SES as compared with middle incomes, while providers were less likely to deliver TS to autistic youth from low SES backgrounds as compared with youth who were not from low SES backgrounds. These findings support literature suggesting that child characteristics are broadly associated with the receipt of ASD-related services (56, 57), and provide a greater understanding of the relation between child characteristics and treatment practice sets reported to be utilized in community care settings. Future research on provider’s practice use decision-making may better elucidate these findings.

### Limitations and future directions

While this study advances the existing services research on community-based care for autistic youth experiencing common co-occurring difficulties, limitations warrant noting. The amount of time the survey took to complete varied by provider response and thus could have been quite long for some providers (e.g., those who serve autistic youth who are experience any of the common co-occurring challenges focused on in this survey). Further, the UCAS survey was not programmed to present survey items in a counterbalanced manner. Thus, respondent fatigue may have occurred.

Despite being the largest and most geographically distinct transdisciplinary sample to date, this sample may not be representative of all providers delivering services to autistic youth with common co-occurring problems across the United States. Future studies involving a more representative sample of providers in the U.S. would be an ideal next step for evaluating the landscape of services reported to be delivered to autistic youth. Moreover, providers were not asked about their use of practices unless they indicated that they were somewhat to very familiar with the practice. Thus, not all providers were presented the opportunity to report on their use of all transdisciplinary practices included in the UCAS survey inventory. Additionally, because the UCAS survey was a self-report measure, the data may not reflect the practices that providers are *actually* delivering in community care settings. Further, quality of services or practice fidelity were not measured. Providers may have reported use of strategies in a specific practice set but have limited adherence to the practice components, thereby negatively impacting service quality. Observational research indicates that therapists in community mental health settings frequently implement several practices consistent with research-based behavioral methods (e.g., positive reinforcement) for autistic children, but that they are implemented with low to moderate extensiveness (i.e., frequency/thoroughness) (27). Future research should evaluate fidelity and quality of providers’ practice set delivery to determine what autistic youth are receiving in community-based settings despite methodological challenges (28). Finally, providers were asked to report on their use of specific strategies for their autistic clients but were not asked to specify whether they utilized the strategies with all clients or only a subset. For example, providers who deliver treatment to autistic youth with and without co-occurring IDD may have indicated their use of specific strategies, such as CBTS, but may not use this set of strategies with the clients on their caseload who had co-occurring IDD. Despite these limitations, this study has important strengths. We focused on individual discrete practices rather than treatment packages and each practice element was defined with concrete definitions, examples, and limited jargon so that providers across disciplines could share a “common language” when evaluating whether to endorse their use of the practice, rather than being confused or biased by the practice jargon.

## Conclusion

Overall, these results reflect the complex and confounding landscape of community-based services for autistic youth in the United States. Further, these results highlight the numerous provider-, setting-, and child-level factors that interact to impact the provider-reported use of these transdisciplinary practice sets within the existing autism system of care (58, 59). By taking this transdisciplinary, transtheoretical, content-agnostic study approach (e.g., broad provider eligibility, reduced practice jargon, etc.), we provide quantitative evidence to elucidate the complexity of autism service provision in the United States. We hope that stakeholders and affinity groups (e.g., researchers, educators, policymakers, special interest groups, practitioners, etc.) will utilize these results to identify areas of strength and improvement within the service system as well as proximal points of engagement and change with community partners, and develop or investigate the effectiveness of multi-level systemic interventions. Ultimately, my co-authors and I hope that these results will motivate dialogue, research, and action to make necessary changes to the system of care for autistic youth.

Systematic efforts must be made to both effectively disseminate evidence-based ASD practices (60) and facilitate practice use through implementation strategies within usual, community-based settings (61). Utilizing autism-specific evidence-based implementation toolkits within community-based agencies and schools serving autistic youth may assist with identifying ASD practices that are feasible and acceptable for use by providers with varied educational and training backgrounds and professional norms, fit within the service setting, and match the needs of the children and families (62–64). Moreover, implementation toolkits–especially those involving community-engaged approaches–may increase organizational and provider readiness for implementing the use of practices (65) and de-implementing unsupported or problematic practices, thereby increasing the quality of ASD community-based psychosocial services and improving the quality of life of autistic youth with co-occurring conditions.

## Data availability statement

The raw data supporting the conclusions of this article will be made available by the authors, without undue reservation.

## Ethics statement

IRB approval was obtained from the following institutions: San Diego State University, Michigan State University, St. John’s University, Drexel University, Rush University, Stony Brook University. The studies were conducted in accordance with the local legislation and institutional requirements. The participants provided their written informed consent to participate in this study.

## Author contributions

AD contributed to study conceptualization, methodology, project multisite administration, recruitment, data collection, statistical analyses, and manuscript writing. AS contributed to statistical analysis and manuscript writing. LM, CK, LS, AW, EC, and ML contributed to study conceptualization, methodology, project administration, recruitment, data collection, results interpretation, and reviewing and editing of the manuscript. ML led conceptualization of statistical analyses within the manuscript. All authors contributed to the article and approved the submitted version.

## Funding

This work was supported by the Adelphi University Center for Health Innovation, Pershing Charitable Trust, Brian Wright Memorial Autism Fund, and by funding through the National Institute of Mental Health (K01MH093477 [PI: Drahota] and R01MH110585 [PI: Lerner]), Eunice Kennedy Shriver National Institute of Child Health and Human Development (K23HD087427 [PI: Kerns]), Michael Smith Foundation for Health Research Scholar Award (SCH-2021-1709 [PI: Kerns]), and Simons Foundation (SFARI #381283 [PI: Lerner]).

## Conflict of interest

The authors declare that the research was conducted in the absence of any commercial or financial relationships that could be construed as a potential conflict of interest.

## Publisher’s note

All claims expressed in this article are solely those of the authors and do not necessarily represent those of their affiliated organizations, or those of the publisher, the editors and the reviewers. Any product that may be evaluated in this article, or claim that may be made by its manufacturer, is not guaranteed or endorsed by the publisher.
